# Deep learning applications in parasite microscopy: A scoping review

**DOI:** 10.1371/journal.pntd.0014774

**Published:** 2026-10-07

**Authors:** Rose Mary Maniraj, Emelia Osman, Siti Norul Huda Sheikh Abdullah, Aishah Hani Azil, Mohd Amierul Fikri Mahmud

**Affiliations:** 1 Department of Parasitology and Medical Entomology, Faculty of Medicine, Universiti Kebangsaan Malaysia, Kuala Lumpur, Malaysia; 2 Center for Cyber Security, Faculty of Information Science and Technology, Universiti Kebangsaan Malaysia, Bangi, Selangor, Malaysia; 3 Institute for Public Health, National Institutes of Health, Ministry of Health Malaysia, Shah Alam, Selangor, Malaysia; Wellcome Sanger Institute, UNITED KINGDOM OF GREAT BRITAIN AND NORTHERN IRELAND

## Abstract

**Background:**

Parasitic diseases remain a significant contributor to the global burden of disease and conventional microscopy remains the gold standard for parasite detection. However, it is labour-intensive and dependent on skilled personnel, leading to variability in diagnostic accuracy. Recent advances in deep learning have enabled automated analysis of microscopy images, offering potential improvements in diagnostic efficiency and consistency.

**Methodology/principal findings:**

This scoping review aimed to map current evidence on deep learning applications in parasite microscopy for human clinical diagnosis. A systematic search of seven electronic databases was conducted for studies published between January 2015 and July 2025. Eligible studies included primary research that applied deep learning models to parasite microscopy images and reported performance metrics. A total of 118 studies were included. Image classification was the most commonly reported task, followed by object detection, while image segmentation was less frequently investigated. A wide range of architectures was used, including convolutional neural networks (CNNs), YOLO-based detection models and hybrid approaches. Most studies reported high performance across tasks, however, direct comparison was limited due to heterogeneity in datasets, evaluation metrics and experimental designs. In addition, most studies relied on internal validation using subsets of the same dataset, with limited external validation. Among the studies included in this review, several proposed implementation strategies. However, relatively few reported implementation or evaluation in real-world clinical or diagnostic laboratory settings.

**Conclusions/significance:**

Deep learning demonstrates substantial potential for automated parasite detection in microscopy. Among the studies included in this review, most deep learning models were evaluated under experimental conditions, with relatively few reporting implementation or evaluation in clinical or diagnostic laboratory settings. Key challenges include dataset heterogeneity, lack of standardised evaluation and limited real-world validation. Future research should prioritise external validation and implementation-focused studies to support integration into clinical and laboratory workflows.

## Author summary

Parasitic infections continue to affect millions of people worldwide, particularly in regions with limited healthcare resources. Diagnosis often relies on microscopy, which requires trained personnel and can be time-consuming and variable in accuracy. In recent years, deep learning has emerged as a promising tool for automating parasite detection in microscopy images. In this study, we reviewed current research on deep learning applications in parasite microscopy. We found that many models can detect and classify parasites with high accuracy under controlled conditions. However, most studies used pre-existing datasets and did not test their models in real clinical settings. In addition, differences in study design and evaluation methods make it difficult to compare results across studies. Our findings suggest that while deep learning has strong potential to support parasite diagnosis, further work is needed to ensure these systems are reliable and practical for real-world use. Future studies should focus on testing models in clinical environments and developing standardised approaches to improve comparability and implementation.

## 1. Introduction

Parasitic diseases continue to pose a major global health challenge, particularly affecting populations in low- and middle-income countries where access to timely diagnosis may be limited. Malaria alone caused an estimated 263 million cases and nearly 600,000 deaths in 2023, with the highest burden seen in sub-Saharan Africa [1]. Early and precise diagnosis is essential for effective treatment and controlling transmission. Traditional light microscopy has long been considered the gold standard for detecting parasites in blood smears, stool samples and tissue samples because it is relatively inexpensive and widely available [2–4]. However, this method is labour-intensive, requires skilled personnel and is subject to inter-observer variability, which can affect diagnostic consistency and throughput, particularly in high-burden or resource-constrained settings [5].

In recent years, digital pathology and artificial intelligence (AI) have provided new opportunities to address these diagnostic challenges. Deep learning, a subset of AI, has demonstrated strong performance in medical image analysis tasks, including tumour detection, retinal disease screening and radiological interpretation [6]. In parasitology, deep learning approaches particularly CNNs and object detection frameworks have been increasingly applied to automate parasite detection, species classification and quantification in microscopy images [7,8]. These approaches enable automated feature extraction and have the potential to improve diagnostic efficiency while reducing reliance on manual interpretation. Early studies have reported promising results, with some deep learning-based systems achieving performance comparable to trained microscopists and enabling rapid or near real-time analysis, including in field-based settings [9–11].

Despite these advances, the current body of evidence remains fragmented. Studies vary widely in terms of model architectures, datasets, evaluation metrics and experimental designs, making direct comparison of reported performance challenging [12]. In addition, most studies rely on internal validation using pre-existing datasets, with limited external validation across diverse laboratory conditions, staining techniques or patient populations, raising concerns regarding generalisability [13]. Although several studies included in this review proposed implementation strategies, including integration into laboratory workflows, mobile platforms and cloud-based systems, relatively few reported implementation or evaluation in real-world clinical settings and key considerations such as usability, scalability and regulatory requirements were often not addressed.

Furthermore, while previous reviews have examined artificial intelligence in parasitology, many have combined conventional machine learning and deep learning approaches or focused on specific parasites or applications. A comprehensive synthesis focusing specifically on deep learning in parasite microscopy across multiple parasite types and computer vision tasks remains limited. This lack of focused synthesis creates uncertainty regarding the maturity, comparability and clinical readiness of existing deep learning–based diagnostic systems.

To address this gap, this scoping review systematically maps the current evidence on deep learning applications in parasite microscopy for human clinical diagnosis. Specifically, this review aims to: (i) identify and characterise deep learning models developed for parasite detection, classification and segmentation in microscopy images; (ii) summarise their reported diagnostic performance and (iii) examine reported and proposed implementation strategies for integrating deep learning models into clinical and laboratory workflows, including real-time and near real-time applications, while assessing the extent to which these approaches have progressed beyond proof-of-concept towards real-world evaluation. By synthesising these aspects, this review aims to provide a clearer understanding of current capabilities, limitations and future directions for the translation of deep learning technologies into clinical parasitology practice.

## 2. Methods

### 2.1 Study design

This study was conducted as a scoping review to map the existing evidence on the application of deep learning approaches in parasite microscopy for human clinical diagnosis. A formal review protocol was not prospectively registered; however, the methodological approach was established prior to conducting the review to ensure transparency and reproducibility. This scoping review was conducted following the methodological framework initially proposed by Arksey and O’Malley [14] and subsequently refined by Levac et al. [15] and the Joanna Briggs Institute (JBI) [16]. To ensure comprehensive and transparent reporting, the review also adhered to the Preferred Reporting Items for Systematic Reviews and Meta-Analyses extension for Scoping Reviews (PRISMA-ScR) Checklist [17]. This approach encompasses five key stages: identifying the research question; study selection; collating, summarizing and reporting the results; identifying relevant studies and charting the data. These stages provide a structured process to map the existing evidence and identify knowledge gaps within a broad research area.

### 2.2 Identifying the research question

This scoping review aimed to address the following research questions (RQs):

RQ1: What deep learning approaches have been developed and applied for parasite microscopy in human clinical diagnosis?
RQ2: What performance metrics have been reported for these deep learning models?
RQ3: What implementation pathways have been proposed, piloted or evaluated for translating deep learning models into clinical or laboratory workflows?

To ensure alignment with the research questions, the Population, Concept and Context (PCC) framework was used to guide the study selection process (Table 1). The PCC framework, recommended by the JBI for scoping reviews, facilitates the systematic identification of relevant studies and ensures consistency between the research questions and the eligibility criteria.

**Table 1:** The Population, Concept and Context addressed in this scoping review.

PCC Framework	Determinants	Inclusion Criteria	Exclusion Criteria
Population	Parasite microscopy for human clinical diagnosis	• Studies involving detection, classification, segmentation or quantification of human parasites using microscopy images	• Not involving human parasite microscopy • Not based on microscopy images
Concept	Development and evaluation of deep learning models in parasite microscopy	• Application of deep learning architectures in parasite microscopy • Reporting of performance metrics	• No application of deep learning in parasite microscopy • Performance metrics not reported
Context	Clinical or diagnostic laboratory implementation settings	• Studies conducted in clinical or diagnostic laboratory settings • Studies proposing, piloting or experimentally evaluating implementation strategies	• Non-clinical research, unrelated to diagnostic application • No description of implementation or intended clinical use

### 2.3 Search strategy

This review identified full research articles published in English that investigated the application of deep learning in parasite microscopy through a structured literature search. The search period was restricted to January 2015 to July 2025, as the widespread adoption of deep learning in biomedical image analysis began around 2015 following major advances in CNNs. Restricting the search to this period ensured that the review captured developments relevant to modern deep learning-based microscopy.

Seven electronic databases were searched: PubMed, Scopus, Web of Science (WoS), IEEE Xplore, SpringerLink, ScienceDirect and ACM Digital Library. These databases were selected to ensure comprehensive coverage of both biomedical and computational research domains. PubMed and WoS primarily index biomedical and clinical literature, whereas IEEE Xplore, ACM Digital Library and SpringerLink cover engineering and computer science research. Scopus and ScienceDirect were additionally included to broaden interdisciplinary coverage, despite partial overlap in indexed content.

The search strategy was developed based on key concepts derived from the research questions. Corresponding Medical Subject Headings (MeSH) terms were identified in PubMed and additional free-text keywords and synonyms were generated to maximise search sensitivity. These MeSH terms and keywords were combined using Boolean operators (“OR” and “AND”). An overview of the search concepts, corresponding MeSH terms and keywords is presented in Table 2. The complete electronic search strategies for each database, including all Boolean operators applied, are provided in S1 Table to ensure transparency and reproducibility.

**Table 2:** MeSH Terms and Free-Text Keywords used in the literature search.

Category	MeSH Terms	Free-Text Keywords
Parasite	parasites, parasitic disease	helminth, nematode, trematode, cestode, protozoa, ova, cysts
Deep Learning Application	deep learning, artificial intelligence, machine learning	lightweight deep learning, convolutional neural network, computer vision, image classification, object detection, image segmentation, image processing
Clinical Laboratory Implementation	clinical laboratory techniques, microscopy, diagnosis	clinical parasitology, medical laboratory diagnosis, blood smear, blood film, direct smear, microscopy images, parasite images

### 2.4 Eligibility criteria

Studies were selected based on predefined inclusion and exclusion criteria aligned with the objectives of this scoping review. Eligible studies included primary research that investigated the application of deep learning approaches to parasite microscopy images for human clinical diagnosis. These studies involved tasks such as parasite detection, classification, segmentation or quantification using microscopy images. Studies were excluded if they did not involve human parasite microscopy, did not apply deep learning methods or did not report performance metrics relevant to the evaluation of the proposed models. Studies that did not provide sufficient information to identify the deep learning model used or its reported performance were also excluded. Publications that were review articles, editorials, book chapters, conference abstracts or other non-primary research outputs were also excluded, as these sources generally do not provide sufficient methodological and experimental details for data extraction. To ensure conceptual consistency across the included studies, approaches based solely on conventional machine learning using handcrafted features and traditional image processing techniques without deep learning components were excluded.

### 2.5 Study selection

Following the database searches, all retrieved records were compiled and duplicate entries were removed prior to the screening process. Duplicate entries referred only to repeated records of the same publication retrieved from multiple databases. Studies were not excluded solely because they analysed the same study population or image dataset, provided they met the predefined eligibility criteria and represented separate primary research articles.

The study selection procedure was conducted in two sequential stages: title and abstract screening followed by full-text review. Titles and abstracts were independently evaluated by two reviewers (RM and EO) to determine their relevance to the objectives of the review. During this stage, studies were initially assessed based on the primary inclusion criterion, defined as the application of deep learning approaches to parasite microscopy images for human clinical diagnosis. Articles that satisfied this primary criterion and appeared relevant to the population and context of the review were retained for full-text assessment.

During the full-text screening stage, articles were evaluated against all predefined inclusion and exclusion criteria described in Section 2.4. Only studies that fulfilled all inclusion criteria and none of the exclusion criteria were included in the final review. Full texts were independently evaluated by two reviewers (RM and EO) and discrepancies between reviewers were resolved through discussion. Where consensus could not be reached, a third reviewer (AHA) made the final decision.

In addition to the database search, backward citation tracking was performed to identify additional relevant studies that may have been missed during the primary search process. Relevant articles were identified by examining the reference lists of all included studies and the cited references were retrieved using Google Scholar. Each potentially relevant article identified through this process was subsequently screened using the same inclusion and exclusion criteria applied during the primary study selection stages. Studies identified through backward citation searching were included if they met the eligibility criteria, irrespective of whether similar datasets had been used in other included studies. This additional step was conducted to enhance the comprehensiveness of the literature search and to ensure that relevant studies cited by the included articles were not overlooked. The overall study selection process was documented using a PRISMA flow diagram, which presents the number of records identified, screened, excluded and included at each stage of the review.

### 2.6 Data extraction

Following study selection, relevant data were extracted from the included studies and recorded in a Microsoft Excel spreadsheet. A standardised data charting form was developed and piloted by the review team prior to data extraction to ensure consistency in the variables recorded (S2 Table). The data extraction sheet captured key study characteristics, including the parasite investigated and corresponding reference, the deep learning model used, dataset size and reported performance metrics. Data extraction was conducted independently by two reviewers (RM and EO) using the standardised data charting form. Extracted information was cross-checked for consistency and any disagreements were resolved through discussion with a third reviewer (AHA). In this review, no critical appraisal of individual sources of evidence was performed, as it was beyond the scope of the study.

### 2.7 Collating, summarizing and reporting the results

The extracted data were synthesised using a descriptive approach to map the application of deep learning models in parasite microscopy. Studies were synthesised at the publication level rather than the dataset level. Consequently, multiple publications based on the same publicly available dataset were retained when they represented separate primary studies that evaluated different deep learning models, analytical objectives or reported outcomes. Each included study was categorised according to its primary computer vision task, namely image classification, object detection or image segmentation. Performance metrics reported in the studies were also recorded and grouped according to the corresponding task type to facilitate structured comparison across studies. Within each task category, studies were further summarised based on the parasite species investigated, deep learning architecture applied, dataset size and reported performance metrics. The reported performance indicators included commonly used evaluation measures such as accuracy, precision, recall, F1-score, sensitivity, specificity, area under the receiver operating characteristic curve (ROC-AUC) and mean average precision (mAP), depending on the computer vision task addressed. However, direct comparison of performance metrics across studies should be interpreted with caution, as substantial variability existed in dataset sizes, class distributions, parasite species, imaging protocols and experimental designs. Due to this methodological heterogeneity, the extracted performance metrics were synthesised descriptively rather than statistically pooled and no formal meta-analysis was conducted.

## 3. Results

### 3.1 Study selection

The study selection process followed the Preferred Reporting Items for Systematic Reviews and Meta-Analyses extension for Scoping Reviews (PRISMA-ScR) framework. The results of the literature search and detailed study selection process are summarised in the PRISMA-ScR flow diagram (Fig 1). The literature was systematically organized using EndNote version 21.5 (Clarivate, London, UK). A total of 1034 records were initially identified from seven electronic databases, comprising PubMed (n = 159), Scopus (n = 319), WoS (n = 269), IEEE Xplore (n = 15), SpringerLink (n = 119), ScienceDirect (n = 147), ACM Digital Library (n = 6). After removal of duplicate records (n = 179), 855 studies remained and were screened based on titles and abstract.

**Fig 1:**
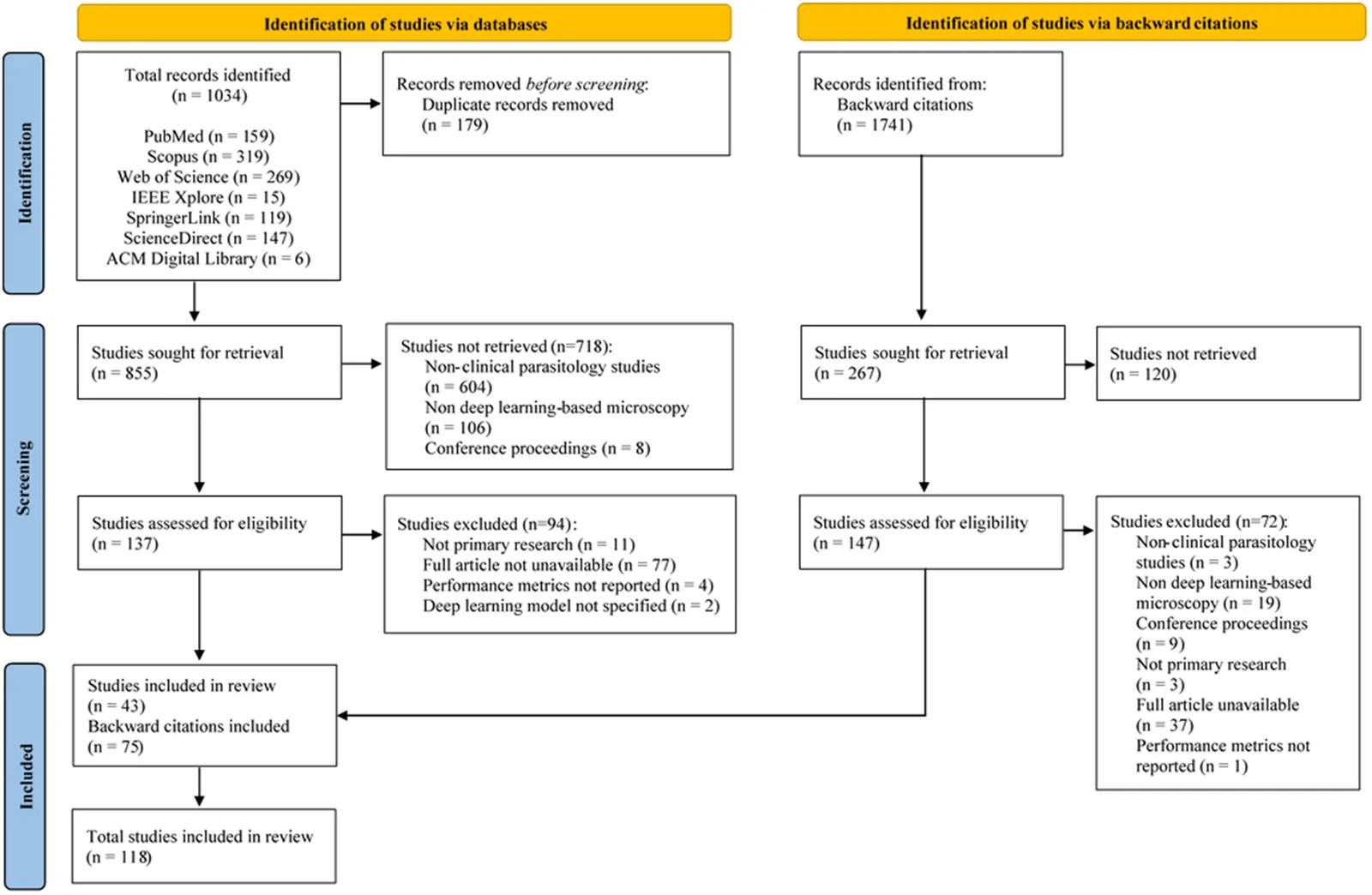
PRISMA-ScR flow diagram [18].

Although seven databases were searched over a ten-year period, the number of included studies was limited due to the strict eligibility criteria applied. During the screening stage, 718 studies were excluded for not meeting the inclusion criteria. The primary reasons for exclusion included non-clinical parasitology studies (n = 604), studies not employing deep learning-based

microscopy approaches (n = 106) and conference proceedings (n = 8). The remaining 137 articles were assessed for full-text eligibility. Following full-text review, 94 articles were further excluded, including non-primary research articles (n = 11), full-text articles unavailable (n = 77), studies not reporting performance metrics (n = 4) and studies where the deep learning model was not specified (n = 2). After the application of all eligibility criteria, 43 studies identified through database searching were included in the review. In addition, backward citation searching identified 1,741 records. After the removal of duplicate records (n = 120), the remaining records underwent screening and eligibility assessment. Of these, 75 studies met the inclusion criteria and were included in the review.

### 3.2 Computer vision task distribution

The included studies were categorized according to the primary computer vision task performed by the deep learning models, namely image classification, object detection and image segmentation. These tasks reflect the main functional roles of computer vision systems in parasite microscopy, ranging from identifying the presence of parasites to localizing them within microscopic images. Among the 118 study entries summarized in Table 3, image classification was the most frequently represented computer vision task (n = 69), followed by object detection (n = 43), while image segmentation was the least commonly reported task (n = 6). Table 3 provides an overview of the included studies, summarizing the parasites investigated, deep learning models applied, dataset size and reported performance metrics. In this table, the dataset size (n) refers to the total number of image samples reported by the original study. To improve transparency, the image unit associated with each dataset is reported where available, including red blood cell (RBC) images, image patches, fields of view (FOVs),

**Table 3:** Summary of included studies by computer vision task.

Ref.	Parasite *	Selected Model	Dataset (n, image unit) †	Reported Performance Metrics (%) ‡
**IMAGE CLASSIFICATION**				
[19]	*Plasmodium* spp.	SNN + CNN	27, 558 RBC images + 517 thin blood smear FOVs	A: 75.39; Sen: 17.90; Spe: 87.04; PPV:22.85; NPV:83.96
[20]	*Plasmodium* spp.	Pretrained CNN (VGG-19)	27,558 RBC images	A: 95.56; F1: 96.45; Sen: 96.65; Spe: 95.25; AUC: 95.45
[21]	*Plasmodium* spp.	Falcon (DCNN)	27,558 RBC images	A: 92.94; P: 93; R: 93; F1: 93
[7]	*Plasmodium* spp.	EfficientNet-B2	27,558 RBC images	A: 97.57; P: 96.59; R: 98.62; F1: 97.55; AUC: 99.21
[22]	*Plasmodium* spp.	CNN	27, 558 RBC images	A: 89.0; P:89.0; R: 89.5; F1: 89.0
[23]	*Plasmodium* spp.	Ensemble learning model combining VGG16, VGG19 and DenseNet201	27,558 RBC images	A: 97.92; P: 97.91; R: 97.92; F1: 97.92
[24]	*Plasmodium* spp.	MCNN	27,558 RBC images	A: 99.24; P: 98.59; F1: 98.94; Sen: 98.48; Spe: 99.29; MCC: 97.80
[25]	*Plasmodium spp.*	DarkNet-53 + DenseNet-201	27,558 RBC images	A: 99.67; P: 99.73; Sen: 99.61; Spe: 99.73; F1: 99.67; MCC: 99.34
[26]	*Plasmodium* spp.	OSICNN	27,558 RBC images	A: 98.3; Sen: 97.68; Spe: 98.19; P: 98.18; F1: 97.93; MCC: 97.66
[27]	*Plasmodium* spp.	Custom CNN (CLR-triangular2)	27,558 RBC images	A: 97.30; P: 97; R: 97; F1: 97; AUC: 97.04
[28]	*Plasmodium* spp.	Ensemble EfficientNetB0	27,558 RBC images	A: 98.29; R: 98.82; P: 97.74; F1: 98.28, AUC: 99.76
[29]	*Plasmodium* spp.	EfficientNetB0 (transfer learning)	27,558 RBC images	A: 94.70; P: 93.87; F1: 94.66; Sen: 95.46; Spe: 93.97
[30]	*Plasmodium* spp.	DACNN	27,558 RBC images	A: 94.79; Sen: 96.32 (infected), 92.91 (uninfected); CK: 89.44
[31]	*Plasmodium* spp.	CNN (3 Conv + 2 FC layers)	27,558 RBC images	A: 96.81; R: 97; P: 97; F1:97
[32]	*Plasmodium* spp.	CNN + LFGWO + SVM	27,558 RBC images	A: 94; P: 97; R: 89.36; F1: 93.75; Spe: 97
[33]	*Plasmodium* spp.	CNN-LSTM-BiLSTM	27,558 RBC images	A: 96.2; P: 97; R: 97; F1: 97
[34]	*Plasmodium* spp.	AOCT-NET (transfer learning CNN)	27,558 RBC images	A: 98.85; P: 98.9; Sen: 98.79; Spe: 98.9
[35]	*Plasmodium* spp.	VGG-19	27,558 RBC images	A: 97.21; Sen: 97.97; Spe: 96.42
[36]	*Plasmodium* spp.	DCNN	27,560 RBC images	A: 95.23
[37]	*Plasmodium* spp.	DCNN	26,188 RBC images	A: 98.9; F1: 98.7; Sen: 98.5; Spe: 98.8; MCC: 97.3; CK: 96.5
[38]	*Plasmodium* spp.	SPCNN with soft attention mechanisms	26,161 RBC images	A: 99.37 ± 0.30; P: 99.38 ± 0.2; R: 99.37 ± 0.2; F1: 99.37 ± 0.2; AUC: 99.95 ± 0.0
[39]	*Plasmodium* spp.	CNN-DELM	26,161 RBC images	A: 99.66; P: 99.69; R: 99.55; F1: 99.62
[40]	*Plasmodium* spp.	Compact Convolutional Transformer	26,161 RBC images	A: 99.25; P: 99.08; R: 99.42; F1: 99.25; AUC: 99.99
[41]	*Plasmodium* spp.	WideResNet	16,500 RBC images	A: 97; P: 93.8; R: 97.71; F1: 95.5; ROC-AUC: 98.6
[42]	*Plasmodium* spp.	VGG19 + attention mechanism	2,058 thick blood smear FOVs	P: 51-83; R:72–85; F1: 60–84; AP:41–78
[43]	*Plasmodium* spp.	Novel CAD scheme (FLANN + SSAE + SMC)	1,182 thick blood smear FOVs	A: 89.10; Sen: 93.90; Spe: 83.10; F1: 94.50
[44]	*Plasmodium* spp.	Deep Belief Network	630 thin blood smear FOVs	A: 96.21; F1: 89.66; Sen: 97.60; Spe: 95.92
[45]	*Plasmodium* spp.	MM-ResNet	1,182 multi-magnification thick blood smear FOVs	A: 98.08
[46]	*P. falciparum*	Custom CNN (Model M4)	79,335 RBC images (26,223 LM; 7,386 AFM; 45,726 FM)	A: 98-99.3
[47]	*P. falciparum*	HPTDL-MPDC	27,558 RBC images	A: 91; P: 89; R: 93; F1: 91
[48]	*P. falciparum*	Stacked CNN	27,558 RBC images	A: 99.96; P: 100; R: 99.93; F1: 99.96
[49]	*P. falciparum*	ResNet50 (Residual Neural Network, 50 layers)	27,558 RBC images	A: 96.7; P: 97; R: 97; F1: 97; AUC: 96.73
[50]	*P. falciparum*	Customized CNN	27,558 RBC images	A: 96.82; Sen: 96.33; Spe: 97.78; P: 96.82; F1: 96.82; MCC: 93.64; CK: 93.64
[51]	*P. falciparum*	Custom CNN	27,558 RBC images	A: 95.28; P: 95.5; Sen: 95.0; Spe: 95.5; AUC: 98.86
[52]	*P. falciparum*	ROENet (ResNet-18 + RVFL + ELM + SNN)	27,558 RBC images	A: 95.73; F1: 95.69; Sen: 94.79; Spe: 96.68
[53]	*P. falciparum*	CapsNet (Imperative Dynamic Routing)	27,558 RBC images	A: 98.82; Sen: 98.36; Spe: 99.30; AUC: 98.82
[54]	*P. falciparum*	Inception-based CapsNet	27,558 RBC images	A: 99.35; F1: 99.36; Sen: 99.57; Spe: 99.12; AUC: 99.73
[55]	*P. falciparum*	ResNet-50	27,558 RBC images	A: 96.17
[56]	*P. falciparum*	ResNet-50 (transfer learning)	27,558 RBC images	A: 95.9; F1: 95.9; Sen: 94.7; Spe: 97.2; AUC: 99.1
[57]	*P. falciparum*	VGG-19 + SqueezeNet (Ensemble-D)	27,558 RBC images	A: 99.51; P: 99.84; F1: 99.50; AUC: 99.92; MCC: 99
[58]	*P. falciparum*	DepthResInceptNet	27,557 RBC images	A: 94.2; P: 96.3; R: 97.0; F1: 96.6; AUC: 97.4
[59]	*P. falciparum*	VGG8	26,159 RBC images	A: 99.30; P: 99.65; F1: 99.33; Sen: 99.66; Spe: 99.42
[10]	*P. falciparum*	Autoencoder-based CNN	26,161 RBC images	A: 99.23; P: 98.92; F1: 99.22; Sen: 99.52; Spe: 99.17
[60]	*P. falciparum*	TL-SGAN	2,500 RBC images	A: 96.6; P: 95.53; F1: 96.33; Sen: 97; Spe: 96.2; AUC: 98
[61]	*P. falciparum*	VGG19-SVM	2,550 thin blood smear FOVs	A: 93.13; P: 89.95; F1: 91.66; Sen: 93.44; Spe: 92.92
[62]	*P. falciparum*	Customized CNN	1,000 RBC patch images	A: 97.7; Sen: 98.3; Spe: 96.9
[63]	*P. falciparum*	DeepMCNN	299 TBFs × 100 FOVs	A: 91; Sen: 92; Spe: 90; PPV: 92; NPV: 90
[64]	*P. vivax*	DTGCN (ResNet50 + GCN)	7,456 patches	A: 98.3; P: 98.5; R: 98.3; F1: 98.3
[65]	*P. falciparum & P. vivax*	CNN-BiLSTM	27,560 RBC images	A: 99.89; P: 99.73; R: 99.89; F1:99.76
[66]	*P. falciparum & P. vivax*	Modified VGG16 CNN (within SSD300-FSRCNN-VGG16 pipeline)	27,558 RBC images; 1,364 thin blood smear FOVs	A: 96.53; Sen: 95; Spe: 98.07; AUC: 99.4
[67]	*P. falciparum &* *P. vivax*	Stacked-LSTM model with attention	27,558 RBC images	A: 99.12; P: 99.59; F1: 99.11; Sen: 98.67; Spe: 99.56; AUC: 99.12
[68]	*P. falciparum* & *P. vivax*	Customized CNN	~27,500 RBC images	A: 98; Sen: 98.5; Spe: 98.8; F1: 98.7
[69]	*P. falciparum &* *P. vivax*	CNN-KNN hybrid model	1,920 blood smear images	A: 89.58
[70]	*P. falciparum,* *P. vivax* & *P. ovale*	CNN	1,920 blood smear images	A: 99.59; P: 95.62; F1: 97.56; Sen: 99.58; Spe: 99.59; MCC: 98.68
[71]	*P. falciparum,* *P. vivax, P. ovale & P. malariae*	Scaled YOLOv4	172 + 830 blood smear images	P: 52; R: 93.8; mAP: 83;
[72]	Protozoa: 6 types	InceptionResNetv2 (transfer learning)	34,298 microscopy images	A: 99.96; P: 0.99; R: 0.99; F1: 0.99
[73]	Protozoa: 6 types	Xception (transfer learning, Adam optimizer)	20,169 microscopy images	A: 96.88
[74]	Protozoa: 6 types	Simplified CNN	4,740 protozoa microscopic images	A: 94; P: 83–99; R: 76–100; F1: 85–99
[75]	*Babesia spp.*	DenseNet121	12,614 RBC images	A: 99; P: 92; R: 100
[76]	*Giardia lamblia*	EfficientNet-B0	1,610 stool microscopy FOVs	A: 96.29; P: 95.99; R: 96.19; F1: 96.07; Spe: 98.21
[77]	*Trypanosoma cruzi*	Res2_SVM	1,983 ROI images	A: 98.48; P: 100; R: 97.20; F1: 98.58; AUC: 100
[78]	*Trypanosoma cruzi*	MobileNetV2 + Single-Neuron Classifier	1,656 image tiles	A: 95.5; P: 97.4; Sen: 93.6; F1: 95.4
[79]	*Toxoplasma gondii*	FCGAN with transfer learning	29,127 multi-magnification + 6,295 macroscopic images	A: 90–94; P: 84–95; R: 92–99; F1: 91–94
[80]	*Leishmania*	LeishFuNet	292 skin scraping FOVs	A: 98.95 ± 1.4; P: 97.91 ± 2.77; F1: 98.92 ± 1.43; Sen: 100; Spe: 98 ± 2.67; AUC: 99 ± 1.33
[81]	*Leishmania*	DenseNet-201	not reported (ultra-thin skin smear images)	A: 99.14; F1: 99.10; Sen: 99.53; Spe: 98.80; PPV: 98.69; NPV: 99.58; AUC: 99.95; MCC: 98.30; CK: 98.29
[82]	*Ascaris lumbricoides* eggs	Custom CNN (3 convolutional layers)	645 helminth egg images	A: 93.33; P: 86.6–100; R: 87.5–93.7
[83]	*Ascaris lumbricoides* eggs	Custom CNN (4 Conv Layers) + OpenCV Moment-Based Detection	400 helminth egg images	A: 90.03
[84]	*Ascaris lumbricoides* & *Taenia saginata* eggs	EfficientNet v2 S	2,327 helminth egg images	Macro P: 98.33; Macro R: 98.25; Macro F1: 98.28
[85]	Intestinal parasite eggs: 15 types	VGG-16	51,919 image patches	CK: Helminth eggs: 94.9 Helminth larvae: 87.8 Protozoa cysts: 92.5
**OBJECT DETECTION**				
[86]	*Plasmodium spp.*	YOLO-mp-3l	3,885 thick blood smear FOVs	mAP@0.5: 93.99
[87]	*Plasmodium* spp.	Fine-tuned YOLOv11m	2210 thick blood smear FOVs	Mean P: 0.828±0.004; Mean R: 78.5 % ±0.2; Mean mAP@50: 86.2±0.3
[88]	*Plasmodium spp.*	Faster R-CNN (ResNet101)	643 thick blood smear FOVs	P: 77.91; R: 89.81; mAP: 94.48
[89]	*Plasmodium spp.*	YOLOv8	333 thick blood smear images	A: 95; Sen: 94; Spe: 93; mAP 95
[90]	*Plasmodium* spp.	Combination of YOLOv5, Transformer & U-Net	1,822 thin blood smear FOVs	AP: 90.8
[91]	*Plasmodium spp.*	YOLOv8s	1,081 thin blood smear images	P: 88.61; R: 84.41; F1: 86.46; mAP@0.5 = 90.31
[92]	*Plasmodium* spp.	YOLO-SPAM++ (YOLOv5-based with attention modules)	555 thin blood smear FOVs	AP: 84-94 (species-dependent) AP50: 92-99
[93]	*Plasmodium* spp*. (+ Babesia, Trypanosoma)*	RT-DETR	29,228 thin blood smear FOVs	Patient-level A: 79.4 (five-class model; ovale/vivax pooled)
[94]	*P. falciparum*	YOLOv4-tiny	5,033 thick blood smear FOVs	R: 95.3; AP: 87.1
[95]	*P. falciparum*	YOLOv3-MOD	3,885 thick blood smear FOVs	Dataset A: A: 99.07; P: 99.17; Sen: 99.79; Spe: 93.33; F1: 99.48 Dataset B: A: 97.46; P: 96.65; Sen: 100.00; Spe: 90.48; F1: 98.30
[96]	*P. falciparum*	CNN	2,967 thick blood smear FOVs	A: 96.89; P: 74.84; Sen: 90.82; Spe: 97.43; AUC: 98.48
[97]	*P. falciparum*	IGMS + Customized CNN	1,819 thick blood smear FOVs	A: 93.46; P: 94.25; F1: 93.40; AUC: 98.39; Sen: 92.59; Spe: 94.33
[98]	*P. falciparum*	YOLOv4-MOD	1,182 thick blood smear FOVs	P: 95; R: 94; F1: 94; Avg IoU: 62.12; mAP: 96.32
[99]	*P. falciparum*	Faster R-CNN ResNet101	903 thick blood smear FOVs	Faster R-CNN:P: 68.6; R: 93.03; F1: 78.97; mAP: 66.09
[100]	*P. falciparum*	RetinaNet (ResNet50 backbone)	8,145 thin blood smear FOVs	AP: 88.5; Sen: 99.5; Spe: 90.0; AUC 99.7
[101]	*P. falciparum*	Faster R-CNN (ResNet-50 backbone)	1,732 thin blood smear FOVs	A: 99.8; AP: 99; Sen: 99.5; Spe: 99.8; F1: 97.6; AUC: 97.9
[102]	*P. falciparum*	Ensemble Weight-Assisted YOLOv5	1,182 thin blood smear FOVs	P: 76; R: 78; mAP@0.5: 79
[103]	*P. falciparum*	RBCNet (U-Net + Faster R-CNN)	695 thin blood smear FOVs	P: 97.54; R: 98.39; F1: 97.94
[104]	*P. vivax*	Cascaded YOLO (YOLOv2 + AlexNet)	2,567 thin blood smear images	mAP: 79.22
[105]	*P. falciparum* & *P. vivax*	Plasmodium VF-Net	5,972 thick blood smear FOVs	A: 92.3
[106]	*P. falciparum* & *P. vivax*	CNN	700 thick blood smear slides	San Juan dataset Sen: 72; Spe: 85; PPV: 77; NPV: 82; A: 80 Santa Clara dataset Sen: 52, Spe: 70, PPV: 52, NPV: 71, A: 64
[8]	*P. falciparum,* *P. vivax, P.ovale, P. malariae* & *P. knowlesi*	Optimized YOLOv4 (YOLOv4-RC3_4)	682 thin blood smear images	P: 59; R: 96; F1: 74; mAP: 90.70
[107]	*P. falciparum,* *P. vivax, P.ovale* & *P. malariae*	YOLOv4	446 thin blood smear FOVs	mAP@0.5 = 90
[108]	Intestinal helminth eggs: 11 types	YAC-Net (YOLOv5n + AFPN + C2f)	13, 200 helminth egg images	P: 97.8; R: 97.7; F1:97.73; mAP@50: 99.13
[109]	Intestinal helminth eggs: 11 types	EfficientNet-B2; YOLOv5	13,200 helminth egg images	P: 79.35; R: 90.77; F1: 84.67
[110]	Intestinal helminth eggs: 11 types	YOLOv7-tiny	11,000 helminth egg images	P: 99.04; R: 97.82; F1:98.9; mAP@50: 98.7
[111]	Intestinal helminth eggs: 11 types	CoAtNet0	11,000 helminth egg images	Avg P: 94; Avg R: 93; Avg A: 93; Avg F: 93
[112]	Intestinal helminth eggs: 5 types	Faster R-CNN (ResNet50 backbone)	2,500 microscopy images	A: 95; P: 97; R: 96; F1: 95; mIoU: 97
[113]	Intestinal helminth eggs: 5 types	YOLOv5	5,883 helminth egg FOVs	P: 93.8–94.4; R: ~ 97; Spe: 91–99.2; mAP50: ~ 97; mAP50-95: 68.5–72.2
[114]	Intestinal helminth eggs: 4 types	AlexNet, ResNet50 (transfer learning)	162 USB microscopy images	A: 87.69–90.77; P: 93.0–96.6
[115]	Intestinal parasites: 34 types	YOLOv4-Tiny	1,773 stool direct smear FOVs	P: 96.25; A: 99.75; F1: 95.66; Sen: 95.08; Spe: 99.89; AUPRC: 96.3
[116]	Helminth eggs: 9 types	YOLOv4	2,083 helminth egg patches	Overall recognition A: 94.41
[117]	*Opisthorchis viverrini*	Custom CNN	200 microscopic images	A: 100; P: 100; R: 100; F1: 100; ROC-AUC: 100; IoU: 69.47
[118]	STH eggs	YOLOv2 + ResNet50	1,180 WSIs	Sen: 76.4–91.9; Spe: 89.7–98.2; PPV: 46.2–78.1; NPV: 95.0–99.6
[119]	STH eggs	SSD MobileNet	2,264 stool microscopy FOVs	Sen: 15.4–71.4; Spe: 61.1–100; PPV: 66.7–100; NPV: 23.9–96.2
[120]	STH eggs	Deep learning-based image analysis algorithm	7,385 FOVs	Sen: 83.3–100; PPV: 69.6–100
[121]	STH eggs	Expert-verified AI using sequential deep learning algorithms	704 WSIs	Sen: 92.2–100; Spe: 98.2–99.3
[122]	STH and *Schistosoma mansoni* eggs	YOLOv7-E6E	7,780 Kato-Katz FOVs	P: 97.9; R: 97.06; F1: 97.47; mAP@IoU0.5: 98.37
[123]	STH and *Schistosoma mansoni* eggs	R-FCN (ResNet-101)	7,780 Kato-Katz FOVs	Pw: 94.9 ± 0.8; Rw: 96.1 ± 2.1
[124]	*Schistosoma haematobium* eggs	RetinaNet (ResNet-101)	205 urine filtration FOVs	Sen: 91; Spe: 85
[125]	Intestinal protozoa: 8 types	SSD Inception v2	343 WSIs	P: ~ 78; R: ~ 83; Sen: 98.88; Spe: 98.11; AP: ~ 81
[126]	*Trichuris trichiura* eggs	SSD MobileNet	1,508 Kato-Katz FOVs	Mean P: 98.44; Mean R: 80.94; Mean F1: 88.50
[127]	Microfilaria: 4 types	SSD MobileNet v2	2,387 multi-magnification FOVs	10x screening model - P: 94.14; R: 91.90; F1: 93.01 40x species differentiation model - P: 95.46; R: 97.81; F1: 96.62
**IMAGE SEGMENTATION**				
[128]	*Schistosoma haematobium* eggs	DeepLabv3-MobileNet v3 (transfer learning)	12,051 urine filtration FOVs	P: 93.75; Sen: 93.75; Spe: 93.94; PA: 99.69; JAC: 84.64; DSC: 86.55
[129]	*Schistosoma haematobium* eggs	U-Net	5,198 urine filtration FOVs	DSC: 44
[130]	*Schistosoma haematobium* eggs	U-Net (ResNet-18 backbone)	869 urine filtration FOVs	A: 95; Sen: 88; Spe: 99; PPV: 98; NPV: 92
[131]	*Plasmodium spp.*	Seg-Sem CNN	1,364 thin blood smear FOVs	A: 99.68; PA: 97.1
[132]	*Leishmania infantum* (amastigote form)	U-Net	150 bone marrow FOVs	A: 80.4; P: 81.5; Sen: 72.2; Spe: 99.6; IoU: 75.2; ROC-AUC: 85.9
[133]	Helminth eggs: 4 types	Modified U-Net	320 Kato-Katz microscopy images	A: 99.8; Sen: 96–97; PPV: 93.4–99.7

* *P. falciparum*: *Plasmodium falciparum*; ***P. vivax***: *Plasmodium vivax*; ***P. ovale***: *Plasmodium ovale*; ***P. malariae***: *Plasmodium malariae*; ***P. knowlesi***: *Plasmodium knowlesi*.

**† AFM**: atomic force microscopy; **FM**: fluorescence microscopy; **LM**: light microscopy; **TBF**: Thick Blood Film.

**‡ A:** Accuracy; **AP**: Average Precision; **AUPRC**: Area Under the Precision-Recall Curve; **Avg**: Average; **CK**: Cohen’s Kappa; **DSC:** Dice Similarity Coefficient; **F1**: F1-Score/ F1 Measure; **IoU**: Intersection over Union; **JAC:** Jaccard Similarity Coefficient; **MCC**: Matthews Correlation Coefficient; **NPV**: Negative Predictive Value; **P**: Precision; **PA:** Pixel Accuracy; **PPV**: Positive Predictive Value; **Pw**: Weighted Precision; **R**: Recall; **Rw**: Weighted Recall; **Sen**: Sensitivity; **Spe**: Specificity.

whole-slide images (WSIs), region of interest (ROI), image tiles, microscopy images and slides. Dataset sizes were extracted as reported by the authors and may represent training datasets, complete datasets, or other study-specific image collections, depending on the information provided in the original publication.

Across the included studies, both protozoan and helminth parasites were represented and a wide range of model architectures were applied, including CNN-based models, YOLO-based detection frameworks and hybrid approaches. Protozoan parasites, particularly *Plasmodium* spp., accounted for the majority of studies in image classification and object detection tasks. Other protozoan parasites investigated included *Babesia* spp., *Giardia lamblia*, *Trypanosoma cruzi*, *Toxoplasma gondii* and *Leishmania* spp. Helminth-focused studies encompassed *Schistosoma haematobium*, *Ascaris lumbricoides*, *Taenia saginata*, *Opisthorchis viverrini*, soil-transmitted helminths (STHs) and other medically important intestinal helminths. Image classification studies predominantly employed CNN-based architectures and their variants, while object detection studies were largely based on YOLO-derived frameworks. Image segmentation studies mainly relied on U-Net-based architectures, although other segmentation approaches were also reported.

Overall, the studies demonstrated strong performance within their respective tasks based on study-specific evaluation metrics. However, these results were obtained under experimental or internal validation settings and do not necessarily reflect validated clinical diagnostic performance. Taken together, the findings summarized in Table 3 highlight the potential of deep learning to support automated parasite detection and analysis across diverse parasitic infections. This includes the capability for scalable implementation, defined here as the ability of these systems to be adapted across different laboratory settings, datasets and parasite species, while accommodating increasing data volumes and enabling integration with automated microscopy platforms without substantial redesign of the underlying model architecture.

#### 3.2.1 Image classification.

Image classification was the most widely adopted computer vision task among the included studies. In this approach, deep learning models are trained to classify microscopic images into predefined categories, such as infected versus uninfected cells or identifying specific parasite species. The majority of the studies focused on the classification of protozoan parasites, particularly *Plasmodium* species, reflecting the global importance of malaria diagnosis. Various CNN architectures were employed, including VGG-19, EfficientNet, DenseNet, ResNet-based models, as well as customised CNN frameworks and ensemble learning models. Several studies also incorporated attention mechanisms, transfer learning strategies, transformer-based architectures and hybrid deep learning frameworks. Many of these studies utilised a publicly available malaria microscopy dataset comprising cell images, evenly distributed between malaria-infected and uninfected cells.

Although high performance was reported across a wide range of architectures, differences in datasets, evaluation protocols and performance metrics precluded direct comparison of model superiority across studies. Beyond malaria, classification approaches were also applied to other protozoan parasites, including *Babesia* spp., *Giardia lamblia*, *Trypanosoma*, *Toxoplasma*, *Trichomonas* and *Leishmania* species. Similar CNN-based architectures were used in these studies, with reported performance metrics generally remaining high despite smaller dataset sizes.

Classification models were also applied to helminth eggs, including those of *Ascaris lumbricoides*, *Taenia saginata*, *Schistosoma haematobium* and other intestinal helminths. These studies employed a range of CNN-based architectures, including customised CNN models, EfficientNet and VGG-based networks, demonstrating the potential of deep learning classifiers for differentiating helminth egg types in microscopy images. However, studies applying image classification to helminth eggs were less frequently reported than those focusing on protozoan parasites. Overall, the findings indicate that deep learning-based image classification models have demonstrated potential for automated parasite identification across a range of study settings and dataset sizes.

#### 3.2.2 Object detection.

Object detection models were the second most common computer vision task identified in this review. Unlike image classification, these models are designed to identify and localize parasites within microscopic images, typically by generating bounding boxes around detected objects. This enables not only detection but also quantification of parasitic burden in microscopy samples. A summary of the detection frameworks used across the included studies is presented in Table 4.

**Table 4:** Distribution of detection frameworks by model type.

Detector Type	Number of Studies	Model Backbones
YOLO-based	18	YOLOv2 + ResNet50, YOLOv3-MOD, YOLOv4, YOLOv4-tiny, YOLOv4-MOD, YOLOv4-RC3_4, YOLOv5, Ensemble Weight-Assisted YOLOv5, YAC-Net (YOLOv5n-based), YOLOv7-tiny, YOLOv7-E6E, YOLOv8, YOLOv8s, YOLOv11m, YOLO-mp-3l, YOLO-SPAM++
SSD-based	4	SSD Inception v2, SSD MobileNet, SSD MobileNet v2
Transformer-based	1	RT-DETR
R-CNN family	7	Faster R-CNN (ResNet50 and ResNet101 backbones), RetinaNet (ResNet50 and ResNet101 backbones), R-FCN (ResNet101 backbone)
Hybrid/ Other	13	AlexNet + ResNet50 (transfer learning), ResNet50 (transfer learning), CNN, Customized CNN, IGMS + Customized CNN, Cascaded YOLO (YOLOv2 + AlexNet), EfficientNet-B2 + YOLOv5, YOLOv5 + Transformer + U-Net, RBCNet (U-Net + Faster R-CNN), Plasmodium VF-Net, CoAtNet0, Deep learning-based image analysis algorithm, Expert-verified AI using sequential deep learning algorithms

YOLO-based frameworks emerged as the predominant object detection approach, reflecting their widespread adoption in parasite microscopy. Hybrid architectures also accounted for a notable proportion of studies, whereas SSD-based, R-CNN family and transformer-based approaches were employed less frequently. Multiple variants of the YOLO family were identified, ranging from earlier architectures such as YOLOv2, YOLOv3, and YOLOv4 to more recent versions including YOLOv5, YOLOv7, YOLOv8, and YOLOv11. Several studies also employed lightweight, modified, or task-specific adaptations, including YOLOv4-tiny, YOLOv7-tiny, YOLOv4-MOD, YOLOv4-RC3_4, YAC-Net, YOLO-mp-3l, and YOLO-SPAM++, reflecting efforts to optimize detection performance and computational efficiency for parasite microscopy applications. These models were applied across a range of parasitic infections, including *Plasmodium* spp., intestinal parasites and soil-transmitted helminths, demonstrating their applicability across different parasite detection tasks. In addition to YOLO-based models, SSD-based models, including SSD MobileNet, SSD MobileNet v2 and SSD Inception v2, were also commonly used. These models were primarily applied in studies involving intestinal protozoa and helminth detection in stool microscopy images, providing an alternative approach for efficient object detection.

A smaller number of studies explored other detection frameworks, including transformer-based models such as RT-DETR for parasite detection. R-CNN family models, including Faster R-CNN, RetinaNet and R-FCN with ResNet-based backbones, were also reported for parasite detection tasks in microscopy images. In addition, several hybrid or alternative models were identified, including models combining YOLO with transformer modules or U-Net architectures integrating YOLO or Faster R-CNN with other deep learning frameworks, transfer learning-based approaches and customized deep learning pipelines. Reported performance varied across studies and experimental settings, with results obtained under study-specific validation conditions rather than external clinical validation. Object detection models were evaluated using a range of task-specific performance metrics, including precision, recall, mean average precision, sensitivity, specificity and F1-score. The predominance of studies involving *Plasmodium* spp. and helminth eggs suggests that object detection frameworks have been most extensively evaluated for these parasite groups, although applications to intestinal protozoa and microfilariae have also been reported.

#### 3.2.3 Image segmentation.

Image segmentation was less frequently reported than classification and object detection among the included studies. Segmentation approaches aim to identify parasite regions at the pixel level, enabling more detailed morphological analysis and quantification. Among the six image segmentation studies identified, segmentation models were applied to both protozoan and helminth parasites, including *Plasmodium* spp. and *Schistosoma haematobium* eggs. A range of architectures were reported, with U-Net-based models being the most frequently used, including standard U-Net, modified U-Net and U-Net integrated with ResNet-18. Other approaches included DeepLabv3-MobileNetV3 and Seg-Sem CNN architectures.

Overall, the reported performance metrics suggest that deep learning-based segmentation models can effectively delineate parasite structures from complex microscopic backgrounds. While U-Net-based architectures were the most commonly used models and generally achieved strong performance, the small number of studies identified limits the generalisability of these findings. The relatively low number of segmentation studies may reflect the substantial effort required to generate detailed pixel-level annotations, resulting in less frequent application of segmentation methods compared with classification and object detection approaches.

### 3.3 Implementation and deployment strategies

In addition to model development, several studies explored potential strategies for implementing deep learning systems in diagnostic workflows. However, most studies did not explicitly describe deployment processes and remained at the proof-of-concept stage, with only a limited number demonstrating real-time or near real-time applications.

A commonly proposed approach involves integrating deep learning models into existing laboratory workflows as assistive tools [87]. In this setting, automated systems function as preliminary screening tools, identifying potentially infected samples for further review by laboratory personnel. For example, some studies described integration into clinical decision support systems (CDSS), where AI-generated outputs are used to assist pathologists in making faster and more consistent diagnostic decisions [19]. A small number of studies reported real-time or near real-time implementations. These included systems integrated with mobile applications or embedded hardware, such as smartphone-based microscopy platforms and single-board computer systems (e.g., Raspberry Pi), enabling immediate analysis of captured images [110]. In addition, one study demonstrated a semi-automated workflow combining whole-slide scanning, AI analysis and expert verification and achieving rapid turnaround times [121]. Several studies also proposed potential deployment pathways, including integration with Internet of Medical Things (IoMT) systems [134], cloud-based platforms [61] and telemedicine frameworks [126]. These approaches allow remote analysis, data storage and expert consultation, particularly in resource-limited settings [127]. Lightweight model architectures were highlighted as suitable for deployment on devices with limited computational capacity, such as mobile phones or portable diagnostic systems.

Despite these advancements, most studies did not progress beyond experimental validation using pre-collected datasets and detailed implementation strategies were often not reported. This indicates that, while deep learning models show promise for automated parasite detection, translation into routine clinical practice remains limited and requires further validation in real-world settings.

## 4. Discussion

### 4.1 Summary of evidence

This scoping review synthesised current evidence on the application of deep learning in parasite microscopy for human clinical diagnosis. Across the included studies, deep learning models demonstrated generally high performance in image classification and object detection tasks, while image segmentation approaches were comparatively underrepresented.

Image classification was the most frequently reported approach, particularly in malaria-related studies, likely reflecting the availability of large, labelled datasets and the relative simplicity of image-level annotations. Among the included studies, a range of CNN architectures was employed, including both customised models and pre-trained architectures such as VGG, ResNet, DenseNet and EfficientNet. The predominance of CNN-based approaches likely reflects their strong performance in image analysis tasks and their ability to automatically learn hierarchical features from microscopy images without the need for handcrafted feature extraction [135]. In addition to classification approaches, object detection models, predominantly based on YOLO architectures, were widely used for parasite localisation and quantification, highlighting their relevance for clinical workflows that require estimation of parasitic burden [110,136]. Image segmentation was less commonly reported and was primarily based on U-Net architectures and related variants. The comparatively small number of segmentation studies suggests that classification and object detection remain the predominant approaches for automated parasite analysis in microscopy images.

A wide range of deep learning architectures was applied across the included studies, including CNN–based models, YOLO-based detection frameworks, transformer-based detectors and hybrid approaches. However, direct comparison of model performance was not feasible due to differences in datasets, evaluation metrics and experimental designs. In addition, comparability across studies is further limited by differences in outcome definitions and evaluation levels, such as whether performance was assessed at the image, object or slide level. Variations in reference standards, including differences in annotation protocols and expert verification, may also influence reported results. Furthermore, the treatment of ambiguous microscopic findings was often insufficiently described. The exclusion of these challenging cases may result in overly optimistic estimates of model performance and limit the generalisability of findings to routine diagnostic settings. Most studies reported high performance for classification tasks, alongside strong precision and mAP for detection models. Nevertheless, these findings should be interpreted in the context of study-specific datasets, validation procedures and experimental settings.

Among the studies included in this review, translation into routine clinical practice remained limited. Although several studies reported pilot implementation, field evaluation, clinical validation or deployment-oriented prototypes in real-world or resource-constrained settings [85,88,89,100,106,109,120,124,128,129], the majority of studies were conducted as proof-of-concept investigations under controlled experimental conditions. While many studies proposed integration into laboratory workflows, mobile platforms, cloud-based systems, automated microscopy devices, or clinical decision-support tools, evidence for large-scale implementation, multicentre evaluation, long-term clinical adoption and integration into routine diagnostic workflows remains scarce among the included studies. These findings suggest an important translational gap between algorithm development and routine clinical implementation within the current body of evidence. Future studies should therefore focus not only on model performance but also on operational feasibility, clinical utility, cost-effectiveness and user acceptance in real-world diagnostic settings.

Previous reviews have explored artificial intelligence and image analysis in parasitology; however, many have included a combination of conventional machine learning and deep learning approaches or have focused on specific parasites or tasks. In contrast, this review provides a focused synthesis of deep learning–based methods across a broader range of parasitic infections and computer vision tasks, while also examining implementation pathways relevant to clinical and laboratory settings. By synthesizing evidence across both proof-of-concept and deployment-oriented studies that met the predefined eligibility criteria, this review provides a more comprehensive understanding of the current state of the field and identifies key gaps that must be addressed to facilitate wider clinical translation and routine implementation.

### 4.2 Limitations

This scoping review has several limitations. Only articles published in English were included, which may have resulted in the exclusion of relevant studies published in other languages. Although multiple databases were systematically searched, relevant studies indexed in other sources may not have been captured. In addition, no formal critical appraisal of study quality or risk of bias was conducted, consistent with scoping review methodology. As a result, the robustness of the included evidence cannot be fully determined.

A further limitation of this review is that, despite using broad taxonomic and parasite-related search terms across seven electronic databases, some eligible studies indexed primarily under disease-specific terminology may not have been retrieved during the initial database search. Although seven electronic databases were systematically searched using predefined search strategies a substantial number of additional eligible studies were identified through backward citation searching. This highlights the value of incorporating backward citation searching to improve the comprehensiveness of scoping reviews in rapidly evolving interdisciplinary fields such as deep learning in parasite microscopy.

Furthermore, this review included only studies reporting identifiable deep learning architectures together with corresponding performance metrics, in accordance with the predefined Concept criterion. Consequently, clinically implemented AI systems for which the underlying deep learning architecture was not reported were outside the scope of this review. Therefore, conclusions regarding the translation of deep learning models into clinical practice should be interpreted within the context of the included studies.

The included studies were also highly heterogeneous in terms of datasets, model architectures and reported performance metrics, limiting direct comparison across studies. In addition, studies reported dataset sizes using different image units, including WSIs, FOVs, blood smear images, image patches and individual cell images. As these image units represent different levels of image acquisition and analysis, comparisons based solely on numerical dataset size should be interpreted with caution.

Furthermore, most studies relied on internal validation using subsets of the same dataset, with limited external or real-world validation. This may lead to overestimation of model performance and restrict the generalisability of the findings. Moreover, variations in annotation procedures, reference standards and evaluation protocols across studies may have further affected the comparability of reported performance metrics.

The reliance on publicly available datasets, particularly for malaria, may also introduce representation bias towards well-studied parasites. Consequently, parasites that are less frequently investigated in the literature may be underrepresented in the current evidence base. Finally, as this review relied on published performance metrics, the potential for publication bias cannot be excluded, as studies reporting favourable results are more likely to be published.

### 4.3 Conclusions

This scoping review demonstrates that deep learning models have substantial potential for automated parasite detection in microscopy, with consistently high performance reported across multiple computer vision tasks and parasite types. However, among the studies included in this review, most deep learning models were evaluated under experimental conditions, with relatively few studies reporting external validation or implementation in routine clinical practice. Key challenges include the lack of standardised and diverse datasets, heterogeneity in performance reporting and evaluation protocols, and the underrepresentation of non-malaria parasites. Among the included studies, implementation strategies remain relatively underdeveloped, with few studies evaluating real-world clinical deployment. Future research should prioritise the development of standardised datasets, rigorous external validation and prospective evaluation in real-world laboratory settings. Addressing these gaps will be essential to enable the safe and effective integration of deep learning technologies into clinical parasitology workflows.

## Supporting information

S1 Table The detailed Boolean search. Boolean search strategies applied across electronic databases to identify studies on parasitic infections using deep learning and image-based diagnostic approaches.
(PDF)

S2 Table Standardized data charting form used for data extraction. The standardized data charting form used during the review process to extract and organize information from the included studies. The form includes the parasite investigated and corresponding reference, study objective, deep learning model, dataset information, image acquisition method, training and hardware information, reported performance metrics, implementation-related information and reviewer remarks.
(XLSX)

S1 Checklist Preferred Reporting Items for Systematic reviews and Meta-Analyses extension for Scoping Reviews (PRISMA-ScR) Checklist. Checklist outlining the reporting items addressed in this scoping review, indicating the corresponding sections of the manuscript where each item is reported.
(PDF)
