# Supplementary material for: Deep learning applications in parasite microscopy: A scoping review
Source: PLoS Negl Trop Dis. 2026 Oct 7;20(10):e0014774. doi: 10.1371/journal.pntd.0014774 (PMC13645086; doi:10.1371/journal.pntd.0014774)
Supplement: Supplementary file 1 [file pntd.0014774.s001.pdf]

S1 Table. The Detailed Boolean Search Strategies for Each Database.

No.	Database	Boolean Search String
1.	PubMed	(helminth* OR nematod* OR trematod* OR cestode* OR protozoa* OR ova OR cyst*) AND ("deep learning" OR "lightweight deep learning" OR "convolutional neural network" OR CNN OR "object detection" OR "image classification" OR "image segmentation" OR "image processing" OR "computer vision") AND ("clinical parasitology" OR "medical laboratory diagnosis" OR "blood smear" OR "blood film" OR "direct smear" OR microscopy OR "microscopy images" OR "parasite images")
2.	Scopus	(helminth* OR nematod* OR trematod* OR cestode* OR protozoa OR ova OR cyst) AND ("deep learning" OR "lightweight deep learning" OR "convolutional neural network" OR "CNN" OR "object detection" OR "image classification" OR "image segmentation" OR "image processing" OR "computer vision") AND ("clinical parasitology" OR parasitolog* OR "medical laboratory diagnosis" OR "blood smear" OR "blood film" OR "direct smear" OR microscop*)
3.	Web of Science	(helminth* OR nematod* OR trematod* OR cestode* OR protozoa* OR ova OR cyst*) AND ("deep learning" OR "lightweight deep learning" OR "convolutional neural network" OR CNN OR "object detection" OR "image classification" OR image segment* OR "image processing" OR "computer vision") AND ("clinical parasitology" OR "medical laboratory diagnosis" OR "blood smear" OR "blood film" OR "direct smear" OR microscop*)
4.	IEEE Xplore	(helminth* OR nematod* OR trematod* OR cestode* OR protozoa* OR ova OR cyst*) AND ("deep learning" OR "lightweight deep learning” OR "convolutional neural network" OR CNN OR "object detection" OR "image classification" OR "image segmentation" OR "image processing" OR "computer vision") AND ("clinical parasitology" OR "medical laboratory diagnosis" OR "blood smear" OR "blood film" OR "direct smear" OR microscop*)
5.	SpringerLink	(helminth* OR nematod* OR trematod* OR cestode* OR protozoa* OR ova OR cyst*) AND ("deep learning" OR "lightweight deep learning" OR "convolutional neural network" OR CNN OR "object detection" OR "image classification" OR "image segmentation" OR "image processing" OR "computer vision") AND ("clinical parasitology" OR "medical laboratory diagnosis" OR "blood smear" OR "blood film" OR "direct smear" OR microscopy OR "microscopy images" OR "parasite images")
6.	ScienceDirect (Elsevier)	("parasite") AND ("deep learning") AND ("microscopy")
7.	ACM Digital Library	("helminth*" OR "nematod*" OR "trematod*" OR "protozoa*" OR "ova" OR "cyst*") AND ("deep learning" OR "lightweight deep learning" OR "convolutional neural network" OR CNN OR "object detection" OR "image classification" OR "image segmentation" OR "image processing" OR "computer vision") AND ("clinical parasitology" OR "medical laboratory diagnosis" OR "blood smear" OR "blood film" OR "direct smear" OR microscopy OR "microscopy images" OR "parasite images")
